# Barriers and incentives for conducting research amongst the ophthalmologists in Sub-Sahara Africa

**DOI:** 10.1371/journal.pone.0197945

**Published:** 2018-10-23

**Authors:** Kazim A. Dhalla, Micheal Guirguis

**Affiliations:** 1 Dr. Agarwal’s Eye Hospital, Dar Es Salaam,Tanzania; 2 CCBRT Hospital, Dar Es Salaam, Tanzania; 3 University of Liverpool–Online Program, Edmonton, Canada; The University of Warwick, UNITED KINGDOM

## Abstract

**Background:**

Research is a critical component amongst the strategies to improve health outcomes of any country. The role of research assumes greater importance in Africa as it carries a larger share of the global burden of diseases, blindness, and low vision. “Vision 2020- the Right to Sight” is a WHO-IAPB collaborated initiative aiming to eliminate preventable blindness by the year 2020. High quality research in eye care is imperative for the initiative to succeed, however, there is a dearth of research in eye care in sub-Saharan Africa in general and specifically in the Eastern, Central, and Southern African (ECSA) region. Identifying the barriers that hamper research in this region is an important step towards the elimination of preventable blindness.

**Methods:**

A structured questionnaire using the SurveyMonkey program was sent to ophthalmologists in the ECSA region and South Africa through their respective regional professional bodies.

**Results:**

Lack of funding, inadequate time and poor research knowledge were the main research barriers while the ability to improve eye health care through research was the main incentive for conducting research. The response rate for South Africa was low, restricting comparisons with other ECSA countries.

**Conclusion:**

The barriers mainly center on financial, human and administrative infrastructure and resources. In spite of the barriers, ophthalmologists in the study region are enthusiastic in research aiming to increase evidence—based knowledge to improve eye health care in line with the goals of “Vision 2020- the Right to Sight” initiative.

## Introduction

Africa carries a large burden of global blindness and visual impairment. By the WHO estimates, 60% of the world’s blind live in Sub Saharan Africa, India, and China. In 1999, WHO in partnership with the International Agency for the Prevention of Blindness (IAPB) launched a global initiative called “Vision 2020- the Right to Sight” targeting to eliminate avoidable blindness, which is preventable in 80% of the cases, by the year 2020 (http://www.who.int/mediacentre/factsheets). Research would, therefore, be an integral part of this initiative if it were to achieve its goals. Though India and China are thriving in ophthalmic research [1], Africa is lagging behind to a large extent [2] with an obvious paucity of scientific literature from Sub Saharan Africa in the major medical databases [3]. Why is there low research productivity in Sub Saharan Africa? If barriers to conducting ophthalmic research exist, with the exception of West Africa, they are unknown for a large part of Sub Saharan Africa. This study explores the barriers and incentives for conducting research amongst the ophthalmologists in Sub Saharan Africa with a specific focus on ophthalmologists in the Eastern, Central and Southern African countries (ECSA) and South Africa.

## Aim

To identify factors that act as barriers for conducting research and factors that encourage research activities amongst the Ophthalmologists in the ECSA region and South Africa.

## Methodology

Cross—sectional survey of Ophthalmologists in the ECSA region (which is formed by the following countries; Tanzania, Kenya, Uganda, Rwanda, Burundi, Democratic Republic of Congo (DRC), Ethiopia, South Sudan, Zambia, Malawi, Botswana, Mozambique, Somalia and Lesotho) and South Africa (SA). West Africa was excluded from the study because a similar study was conducted in the region in 2011 [4]

### Study design

Cross- sectional survey study.

### Inclusion criteria

All ophthalmologists in the ECSA region and SA irrespective of ethnicity and whether in clinical practice, research, administration or retired.

### Exclusion criteria

African ophthalmologists originally from the study region but, currently residing out of the study region.

### Sample size

The study region is estimated to have about 622 Ophthalmologists and distributed as shown in “Table 1”: (www.icoph.org.2012), [5]

**Table 1:** Distribution of Ophthalmologists in the study region.

COUNTRY	OPHTHALMOLOGISTS
Tanzania	34
Kenya	86
Uganda	40
Ethiopia	107
Malawi	8
Zambia	18
Rwanda	13
Burundi	16
DRC	67
South Africa	233
Total	622

Assuming 20% of the participants (124) are not reachable and a response rate of 60%, the sample size was calculated to be 300 as follows:

622‑124=498X60%=298.8roundedto300.

However, the target was to register all the ophthalmologists hence census sample was extracted.

### Survey tool

A structured questionnaire was used to collect data from the participants using the online SurveyMonkey program (www.surveymonkey.com). Some questions were adapted, with the author’s permission, from similar study done in Nigeria, West Africa [4]. The questionnaire was divided into 6 sections having 28 questions as follows; Introduction of the research topic and its importance in the study region, participant consent, personal and professional data, including name, age, and gender, year of postgraduate qualification and place of current residence. Professional data included work description (clinical or academic), place of work, time spent in private practice, additional research qualification and current involvement in research work. Research barriers and incentives were then explored using closed and open- ended questions. Finally, the participants were thanked for taking part in the study. The full questionnaire is accessible from https://www.surveymonkey.com/r/Eye_Research.

### Pilot testing

A URL linked questionnaire was sent to 10 Ophthalmologists outside the study area and were requested to participate in the pilot study. All the respondents found the questionnaire easy to fill and took less than ten minutes to complete and only one respondent felt that some questions were not well worded.

### Ethical issues

The study is extracted from a dissertation for a Master of Science degree (M.Sc) in clinical research with the University of Liverpool (online course). Ethical clearance for the study was granted by the University of Liverpool ethics committee after obtaining permission to conduct the research in the study area from the regional Ophthalmological bodies; College of Ophthalmology of Eastern, Central and Southern Africa (COECSA) and the Ophthalmological Society of Southern Africa (OSSA) respectively.

### Participant recruitment procedure

The COECSA secretariat office sent out electronic mails with the URL link of the questionnaire to all the members. The study was also advertised on OSSA’s web -based monthly newsletter circulated by electronic mail to all the members. Subsequently, 3 follow up reminders were sent to the members requesting them to participate in the study. Additionally, personal emails with two follow up reminders were sent to the chairpersons of individual countries’ Ophthalmological societies requesting them to encourage their members to participate. Participant recruitment started on 1^st^ April 2016 and access to the questionnaire was closed on the 20^th^ of May 2016. It was not possible to know how many ophthalmologists actually got the information.

Consent was taken by asking participants to “tick” the consent box in the questionnaire if they agreed to participate. Furthermore, the action of filling the questionnaire itself was taken as a surrogate for consent. Participant Information Sheet (PIS) was included in the online questionnaire which specified that participation was voluntary with the option of not answering personal questions like name, age and/or gender. Access to the database was restricted to the researchers only thus ensuring complete confidentiality of the participants’ information.

### Data entry process

All responses were stored in the SurveyMonkey program and directly downloaded to the statistical package, SPSS version 21 (IBM SPSS Statistic) in the coded form.

### Data cleaning and analysis

Data cleaning was done by running a frequency distribution of all the variables. A few respondents preferred not to mention their names, age and/ or gender. Questionnaires without the demographic data were included in the analysis as this would not affect the overall results.

Research productivity, defined as the number of research papers published in the 10 year period from 1^st^ January 2005 to 31^st^ December 2014, was the dependent variable. Independent variables included age, gender, number of years in practice, type of the institution i.e. government, non-government or private, the post held i.e. Clinical, academic, both clinical and academic or purely administrative; time spent in private practice, additional postgraduate training in research and pre-defined research barriers. Descriptive statistic was done using the frequency distribution and measure of central tendency appropriate for the data. Inferential statistics was done using the chi-square test for the 2 by 2 nominal variables and Somer’s delta (Somer’s d) test for the ordinal variables. Poisson regression analysis was done to determine the statistical association between the dependent and independent variables. A p value of <0.05 was taken to be statistically significant.

## Results

### Survey response

There were a total of 114 respondents from the region. The response rate was therefore 38%, assuming that the survey questionnaire reached all the 300 potential participants. The country wise distribution of the respondents is given in “Table 2”.

**Table 2:** Distribution of respondents by study region.

COUNTRY	N	%
Tanzania	25	22
Kenya	47	41
Uganda	8	7
Ethiopia	7	6
Rwanda	3	2.6
DRC	1	0.9
Somalia	1	0.9
Botswana	1	0.9
Mozambique	1	0.9
Zambia	2	1.8
Malawi	3	2.6
Lesotho	1	0.9
South Africa	14	12.3
Total	114	99.8

The majority of the respondents, 87.7%, were from the ECSA region.

#### Socio- Demographic description of the population

“Table 3” summarises the socio-demographic description of the study population.

**Table 3:** Socio-demographic description.

Variable	n	%
**Age (Years) N = 106**		
Mean	43.8	
Range	30–64	
**Gender N = 114**		
Male	72	63
Female	42	37
**Number of years in Practice N = 114**		
Mean	10.4	
Range	1–34	
**Job description N = 114**		
Clinical only	29	25.4
Clinical and academic	79	69.3
Others	14	12.3
**Work set up N = 114**		
Governmental organization	72	63.2
Non- governmental organization	42	36.8
**Private practice engagement N = 114**		
None	46	40.4
Part—time	58	50.9
Full- time	10	8.8

A third of the respondents were in their early careers (1–5 years) and 19.3% were fresh graduates. The majority of the clinicians(69.3%) were also involved in academic practice either as university lecturers or teaching younger cadres including residents on attachment and cataract surgeons. Of the 42 respondents working in non-governmental organisations, 18 were working in faith- based and 12 in private organisations.

### Academic and research profile

Respondents’ academic and research profiles and areas of research interest are given in “Table 4” and “Table 5”.

**Table 4:** Academic and research profiles.

PARAMETER	n	%
No. of Scientific papers published in the past 10 years N = 114		
0	38	33
1–5	49	43
>5	27	24
Currently involved in research N = 114		
Yes	66	58
No	48	42
Interested in research N = 114		
Yes	108	94.7
No	6	5.3
Possession of research degree N = 114		
Yes	18	15.8
No	96	84.2

**Table 5:** Broad areas of research interest.

Area of research	N	%
Vitreo-Retinal	26	23.4
Anterior segment	32	28.8
Glaucoma	15	13.5
Pediatric Ophthalmology	15	13.5
Others	23	20.7

A third of the respondents did not have any scientific publication in the past 10 years, 19 (16.7%) had only 1 publication, while, 10 respondents (8.8%) published more than 10 papers in the same duration The number of papers published was statistically significantly related to the participant’s age (p = 0.000) and the number of years in practice (p = 0.000), however, the Spearman correlation coefficient was not very strong in both the cases; Age ρ = 0.437 and years in practice ρ = 0.5. There was no statistically significant association between the number of papers published and possession of a research degree (p = 0.077). 6 participants (5.2%) published at least 20 papers, all of whom had research degrees. One participant with a Ph.D. degree was involved in 40 publications.

Anterior segment included the lens (cataract) and corneal pathologies and others include Oculoplasty (6.3%), community ophthalmology and epidemiology (4.5%), ocular malignancy, uveitis and conjunctival diseases 1.8% respectively, neglected tropical diseases, refractive errors, nutritional diseases and Trachoma 0.9% respectively. Interestingly, there was no mention of Neuro-ophthalmology.

Though 14 participants had research degrees in community health and epidemiology, only 5 had an interest in the field. It is interesting to note that only 1 respondent was interested in trachoma.

### Research barriers

**Fig 1:**
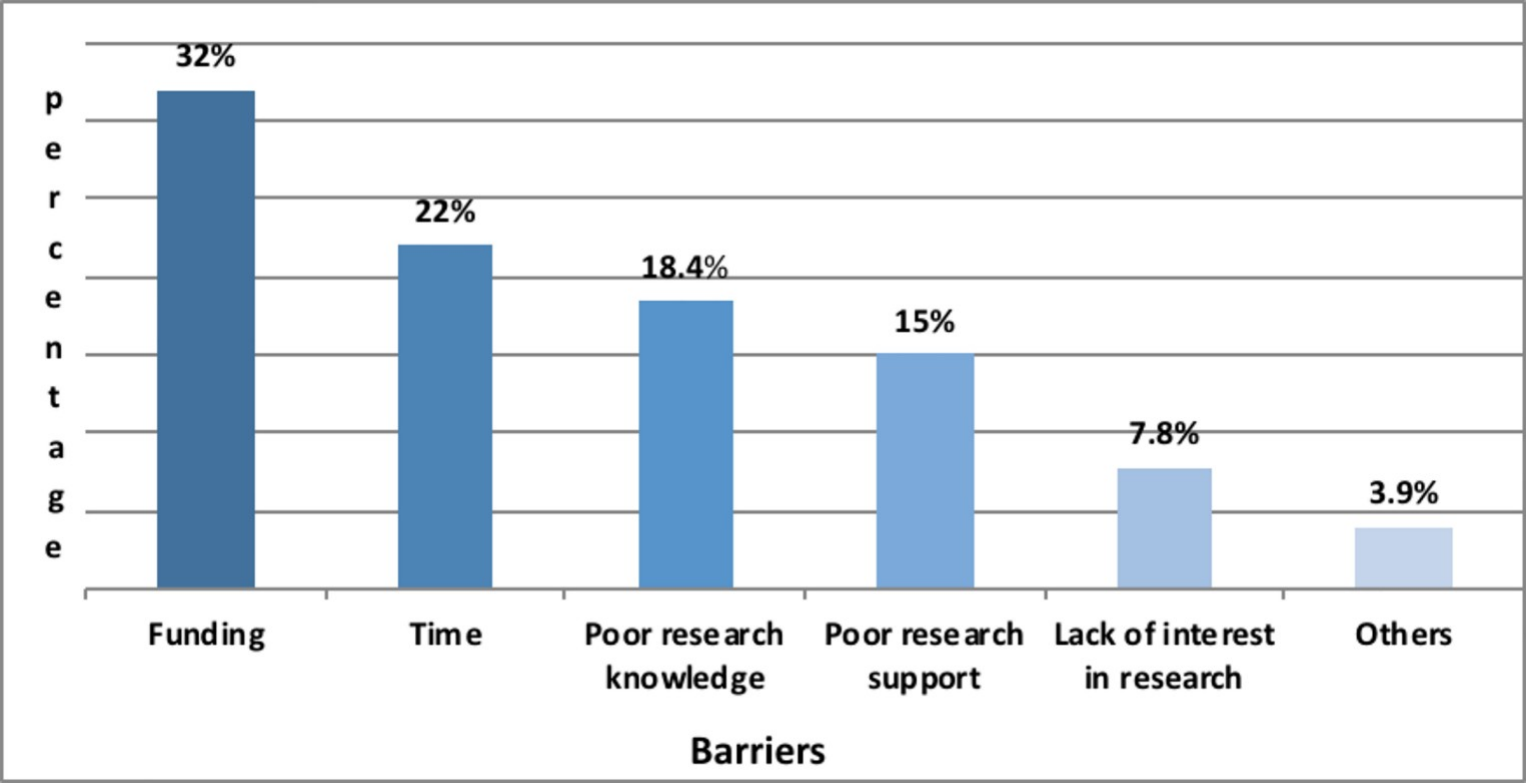
Research barriers.

The majority of respondents, 101/110 (91.8%), felt there were significant barriers to conducting ophthalmic research in Sub Saharan Africa. Fig 1 shows the frequency distribution of the barriers mentioned by the respondents.

#### Funding

The majority of the respondents, 60 (53.6%), cited funding to be the major barrier to conducting research. Non- Governmental Organizations (NGO) stood out to be the most important source of research funding. Nearly a quarter of the respondents used personal funds for research while a third of the participants had never applied for research funding.

#### Time

Half of the respondents had no time for research due to busy clinical commitments. A fifth had dedicated research time, but the majority thought that was not enough. There was no statistically significant relationship between the availability of research time and whether one worked in a government or non-government set up, (p = 0.647).

#### Knowledge

Respondents were asked to assess their knowledge in three broad areas; research process, common statistical software and word processing programs.

More than a half reported good knowledge of all stages of the research; however, statistical skills were poor in a large proportion of participants. Research knowledge was statistically significantly related to having an additional research degree (p = 0.001). ECSA participants reported significantly better statistical skills than the South African peers (p = 0.01). SPSS and EpiInfo were the two commonly known statistical programs, however, the majority of the respondents had poor working knowledge of all the statistical packages.

#### Research support

Research support was assessed on two areas, general research support given at the workplaces and access to electronic resources. Generally, respondents reported poor research support at their workplaces, however, research support is better in academic compared to non-academic institutions (p = 0.016) and access to ethics committees was better in government compared to non-government institutions (p = 0.000).

Though internet was readily available, e- resources, including HINARI was not accessible to the majority of the respondents. There were significant differences between ECCSA countries and South Africa in this area. E- Resources were more accessible to South African respondents (p = 0.045) while HINARI was more accessible to ECSA respondents (p = 0.003).

#### Publication barrier

The majority of the respondents (75/108, 69.4%) felt it was difficult for the African researchers to publish in non-African journals. There was no difference between the ECSA and South African participants (p = 0.108)

#### Factors associated with research output

A multivariate analysis was done using the Poisson regression model to determine the factors associated with research output. “Table 6” summarizes the results.

**Table 6:** Factors associated with research output.

PARAMETER	Chi -Square	p-value	Significance
Academic practice	8.77	0.003	S
Clinical practice only	2.10	0.136	NS
Administrative position	5.12	0.024	S
Years in practice	14.76	0.000	S
Private practice	14.48	0.000	S
Research involvement	47.45	0.000	S
Possession of research degree	34.31	0.000	S
Interest in research	6.27	0.012	S
Knowledge of research process			
Formulating research question	0.346	0.56	NS
Conducting good literature search	4.22	0.04	S
Deciding on study design	4.72	0.03	S
Good statistical skills	3.29	0.07	NS
Academic writing skills	3.18	0.07	NS
Research support			
Presence of research department	43.13	0.000	S
Presence of full- time statistician	87.48	0.000	S
Presence of research assistant	0.97	0.33	NS
Easy access to IRB	22.62	0.000	S
Research support at workplaces	8.50	0.00	S
Access to electronic resources	45.94	0.00	S
Access to HINARI	35.89	0.00	S

S = Factors which are statistically significantly associated with research output

NS = Factors which are not statistically significantly associated with research output.

#### Incentives for conducting research

**Fig 2:**
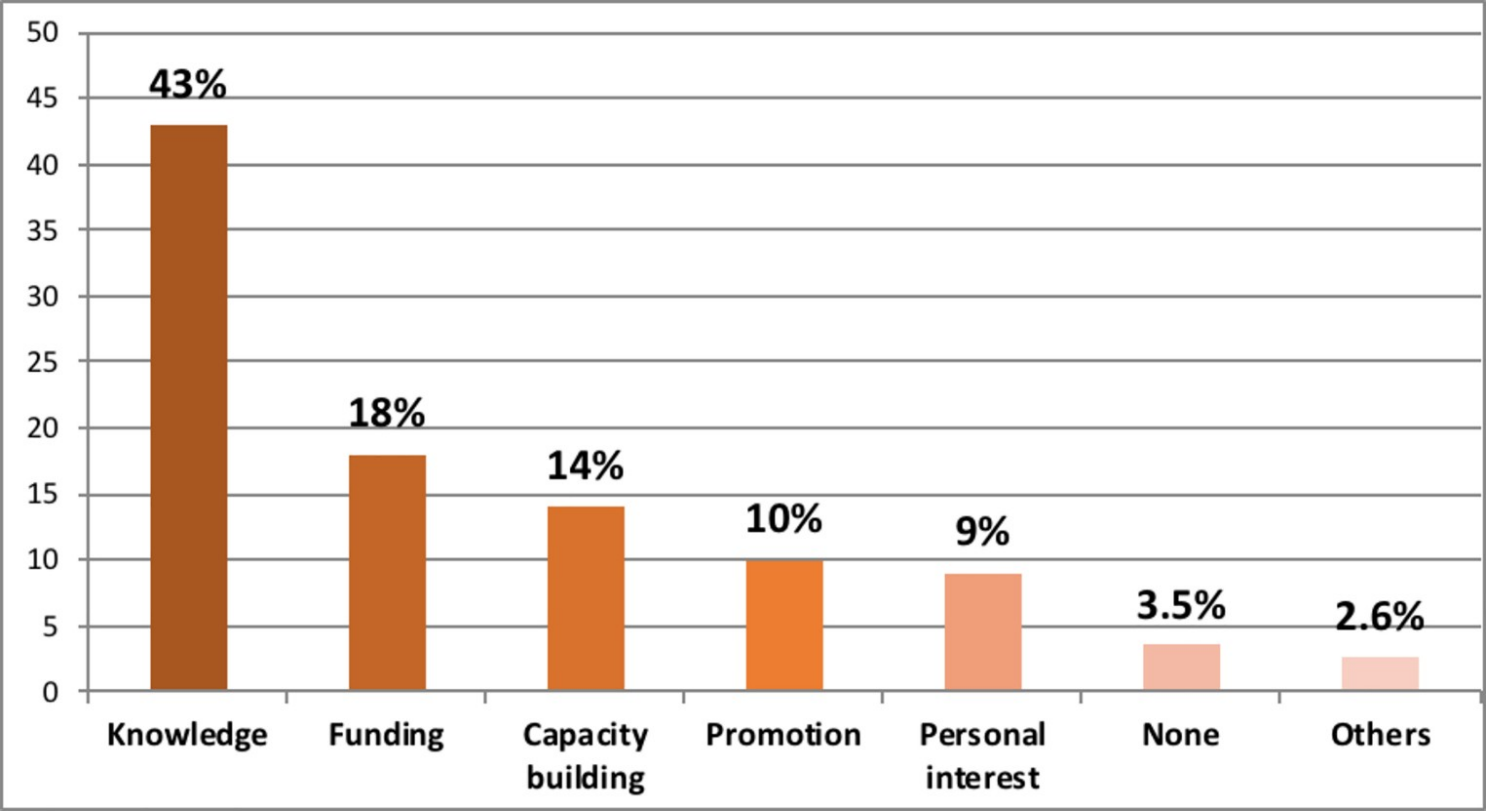
Incentives to conduct research.

Fig 2 gives a frequency distribution of the incentives that drive the participants to conduct research in the region.

The main incentive to conduct research was to expand the existing knowledge base in order to provide evidence- based management of patients. A small proportion of respondents felt there were no incentives for conducting research.

## Discussion

A number of barriers and the associated factors for conducting ophthalmic research in the regions have been identified. Poor funding, inadequate time for research, poor research knowledge and departmental support were the prominent barriers. Research productivity was significantly associated with the academic practice, possession of research degree, research knowledge, research support and access to electronic resources. Contributing to and expanding the existing knowledge base was the main incentive for conducting research amongst the ophthalmologists in the study region. There were no major differences in barriers and incentives between the ECSA and South African participants.

Most of the respondents were involved in research activities and a large majority were interested in research. The high interest in research amongst the ECSA ophthalmologist mirrors that of East African Orthopedic surgeons [6] but strikingly different from the poor research interest amongst the Nigerian Ophthalmologists [4]. The authors of the Nigerian study felt that poor research interest amongst the Nigerian ophthalmologists is due to funding constraints and inadequate knowledge in the research process which feature among the major barriers to conducting research in Nigeria.

### Research barriers

#### Funding

Research funding was one of the main barriers to conducting research. This is consistent with the findings from Nigeria amongst the ophthalmologists [7] and medical specialists [8] and East African orthopedic surgeons [6]. Personal funds were the main source of funding for Nigerian ophthalmologists and medical specialist. Research is an expensive venture and if researchers have to rely on personal funds for research, then this is a great disincentive for conducting good quality, high impact research which also requires funds for publication. It appears that African researchers have not yet explored funding partnerships with the pharmaceutical industry or other corporate sponsors. Standard Chartered Bank, for example, is leading in funding eye care services and training in their novel Seeing is Believing (SiB) project in collaboration with a number of NGOs like the Fred Hollows Foundation, ORBIS and IAPB [9]. African researchers rate government funding last on the list in spite of WHO recommending and governments endorsing the 2% health budget dedication to research.

#### Time

Time constraint featured prominently in our study and appears to be a common barrier across different specialties and regions [10], [11], [12] n. Though it is not practical to separate clinical practice from research work in Africa, a certain number of hours per week could be allocated to clinicians for research work. It was expected that ophthalmologists working in government setups would have dedicated and perhaps more time for research compared to those engaged in private practice. This did not show up in our study.

#### Knowledge

Knowledge of the research process was a significant barrier in our study. Statistical skills appear to challenge a large section of ophthalmologists. Hence, the presence of a statistician in the department was statistically significantly associated with increased research productivity. The decision on selecting an appropriate study design and paper writing skills were also a problem though to a lesser extent. Ophthalmologists with additional postgraduate training in research had good knowledge of all the components of the research process and they had a higher research productivity (p = 0.000). This component does not feature very well in Nigerian studies, however, it appears to be a major barrier amongst the orthopedic surgeons in East Africa as well as Asian doctors. This may be an indicator that there is an inadequate training of research process both at the undergraduate and postgraduate level. Research involvement in medical school appears to have a stronger influence on research productivity [11].

#### Access to academic literature

The majority of the respondents had good internet facilities, however, most of the respondents did not have easy access to electronic academic literature and just about half had access to the Health Inter Network Access to Research (HINARI) program. HINARI was initiated by WHO sponsored private–public partnership in 2002 and offers free access to a large collection of prestigious journals to health institutions in developing countries[13]. In spite of this, 48% of the respondents in our study did not have access to it. This is in contrast to the Nigerian study whereby electronic literature was the main source of scientific information to the ophthalmologists and HINARI is widely accessible to Nigerian researchers [14]. A study on access to electronic scientific knowledge in selected East and West African countries found that more than a third of postgraduate doctors relied on textbooks for information and though internet was generally available, accessibility varied in private and national institutes. Generally, awareness of free online resources, including HINARI was low in the West African compared to East African institutions. HINARI requires an Institutional password and is not accessible to individual researchers [15].

#### Research incentives

The biggest incentive for conducting research amongst the ophthalmologists in our study was to increase evidence—based knowledge in the region. Another important incentive in our study was the ready availability of research funding, which contrasts with the idea of actually looking for funding from donors. Research capacity building and academic promotions also featured as important incentives. Financial gain, fame and international travel to attend and present research findings did not feature at all in our study. It appears that ophthalmologists in this study are fully aware of the fact that research is not a venture for financial growth. Perhaps, there is also an element of altruism as well. Enhancement of knowledge was also the greatest incentive for conducting research amongst the Nigerian ophthalmologist [7] and medical specialists [8], East African orthopedic surgeons [6] and Asian doctors [10]. However, financial gains and fame featured quite prominently in the Nigerian and Asian studies. Capacity building featured as the third most frequent incentive cited which parallels poor research knowledge as a barrier to research productivity. Building research capacity by training the local experts in the research process appears to be a single most important factor that will address both barriers and incentives for research productivity [16].

## Conclusion

A number of barriers have been identified in this study, which appears to hamper research productivity in Sub-Sahara Africa. Dedicated research time, funding and lack of appropriate research skills are the main barriers which, if addressed, will increase research output in the region.

### Study limitations

1. The survey response rate was 38% only which is quite low. This is an inherent limitation of the survey study design [17]. All efforts were made to get as many respondents as possible in the time limit set for data collection. However, the response rate was very good in the ECSA countries where the study is supposed to have more impact.
2. Since a large area of the continent was studied, distribution of the respondents is skewed. This reflects the wide variation of the number of ophthalmologists in the respective countries of the region in general and this could have introduced an element of responder bias.
3. Though comparative analysis of the studied parameters between South Africa and ECSA countries did not show any significant difference, the response rate for South Africa was low, restricting comparisons with other ECSA countries.

### Study strengths

1. This is the first study done on research barriers in the ECSA region and South Africa. The findings are particularly important in the context of the Vision 2020 initiative and will provide a good basis for future research in this area.
2. A large area of Sub Saharan Africa was included in the study and the findings provide a reasonably good reflection of the ground reality in the region.

## Supporting information

S1 Dataset (XLSX)
